# Crosstalk Mechanisms Between HGF/c-Met Axis and ncRNAs in Malignancy

**DOI:** 10.3389/fcell.2020.00023

**Published:** 2020-01-31

**Authors:** Xin Liu, Ranran Sun, Jianan Chen, Liwen Liu, Xichun Cui, Shen Shen, Guangying Cui, Zhigang Ren, Zujiang Yu

**Affiliations:** ^1^Precision Medicine Center, The First Affiliated Hospital of Zhengzhou University, Zhengzhou, China; ^2^Key Laboratory of Clinical Medicine, The First Affiliated Hospital of Zhengzhou University, Zhengzhou, China

**Keywords:** non-coding RNA, HGF/c-Met axis, cancer, crosstalk, mechanism

## Abstract

Several lines of evidence have confirmed the magnitude of crosstalk between HGF/c-Met axis (hepatocyte growth factor and its high-affinity receptor c-mesenchymal-epithelial transition factor) and non-coding RNAs (ncRNAs) in tumorigenesis. Through activating canonical or non-canonical signaling pathways, the HGF/c-Met axis mediates a range of oncogenic processes such as cell proliferation, invasion, apoptosis, and angiogenesis and is increasingly becoming a promising target for cancer therapy. Meanwhile, ncRNAs are a cluster of functional RNA molecules that perform their biological roles at the RNA level and are essential regulators of gene expression. The expression of ncRNAs is cell/tissue/tumor-specific, which makes them excellent candidates for cancer research. Many studies have revealed that ncRNAs play a crucial role in cancer initiation and progression by regulating different downstream genes or signal transduction pathways, including HGF/c-Met axis. In this review, we discuss the regulatory association between ncRNAs and the HGF/c-Met axis by providing a comprehensive understanding of their potential mechanisms and roles in cancer development. These findings could reveal their possible clinical applications as biomarkers for therapeutic interventions.

## Introduction

Cancers are initiated by genetic preconditions and epigenetic alterations, accompanied by various mechanisms, most of which are still unclear (Bach and Lee, 2018). According to Hanahan and Weinberg, these mechanisms are characterized by eight hallmark capabilities which include: sustaining proliferative signaling, evading growth suppressors, activating invasion and metastasis, enabling replicative immortality, inducing angiogenesis, resisting cell death, avoiding immune destruction, and dysregulating cellular energetics (Hanahan and Weinberg, 2011). Despite recent advancements in early diagnosis and personalized treatments, cancer incidence is increasing steadily, and the overall survival rate of patients remains poor (Balani et al., 2017). Therefore, it is crucial to develop a comprehensive understanding of the molecular mechanisms underlying tumor development and progression.

Hepatocyte growth factor (HGF)/c-mesenchymal-epithelial transition factor (c-Met) axis is an essential mediation axis that regulates cellular biological events including cell proliferation, migration and morphogenesis and tumor biological processes such as angiogenesis and drug resistance (Ebens et al., 1996; Arnold et al., 2017). The molecular mechanism of this activation involves gene amplification, overexpression of c-Met and HGF proteins, incremental crosstalk between c-Met and other tyrosine kinases, and MET gene mutation (Zhang et al., 2018). Furthermore, this axis has been generally reported to deregulate in tumor tissues. And its constitutive over-activation, with canonical signaling pathways containing effector molecules such as STAT3/5, PI3K-AKT, and RAS-MAPK activating, which induce malignant phenotype and promoting tumorigenesis, has been implicated in the progression of multifarious cancers (Fukushima et al., 2018). For example, Han et al. found that HGF treatment-induced epithelial-mesenchymal transition (EMT) -like changes and increased the invasive potential of PC-3 cells in human prostate cancer through ERK/MAPK (extracellular-signal-regulated kinase/mitogen-activated protein kinase) and zinc finger E-box binding homeobox 1 (Zeb-1) signaling pathways (Han et al., 2016).

Non-coding RNAs are a cluster of RNA that have non-protein-coding transcripts and are the most common RNA species in eukaryotic cells, including rRNA, tRNA, microRNA, snoRNA, piwi-interacting RNA (piRNA), circRNA, lncRNA, and so on (Slack and Chinnaiyan, 2019). Since the last decade, increasing studies have confirmed that ncRNAs play pivotal roles in various tumors development by targeting diversified downstream genes and signaling pathways and serve as tumor suppressors or stimulators (Higashio et al., 1990; Martianov et al., 2007; De Smet et al., 2015). Among them, miRNAs (small 20- to 25-nucleotide-long RNAs) and lncRNAs (larger than 200 nucleotides long) are the most common molecules that are investigated worldwide. In the recent past, a significant interaction network between HGF/c-Met axis and ncRNAs has been observed in a considerable number of tumors, stressing further the importance of the HGF/c-Met axis in tumorigenesis.

In this review, the crosstalk between the HGF/c-Met axis and ncRNAs (mostly miRNAs and lncRNAs) in common human cancers are summarized. Also, a comprehensive understanding of their potential mechanisms and roles in cancer development are provided. These findings could open a broader horizon for investigators to engage in exploring the molecular mechanisms in cancer development and progression.

## An Overview of HGF/c-Met Axis

HGF, also known as scatter factor, is located on chromosome 7q21 and is a member of the peptidase S1 family of serine proteases, but is short of peptidase activity (Garcia-Vilas and Medina, 2018). It was initially identified as a molecule with the ability to stimulate hepatocyte growth and liver regeneration (Ebens et al., 1996). It is a large multi-domain heterodimeric protein comprising of α-chain (69 kD) and β-chain (34 kD) subunits and is mainly secreted by mesenchymal cells. The α-chain subunit contains an N-terminal hairpin loop and four kringle domains (K1–K4), which are responsible for the high-affinity binding to the c-Met receptor (Higashio et al., 1990; Organ and Tsao, 2011; Chen C. T. et al., 2012). Through cleaving at the Arg494-Val495 bond, it can transform from an inactive single-chain precursor (pro-HGF) to mature HGF after activation by extracellular proteases such as cellular type II transmembrane serine proteases (Fukushima et al., 2018).

**Figure 1:**
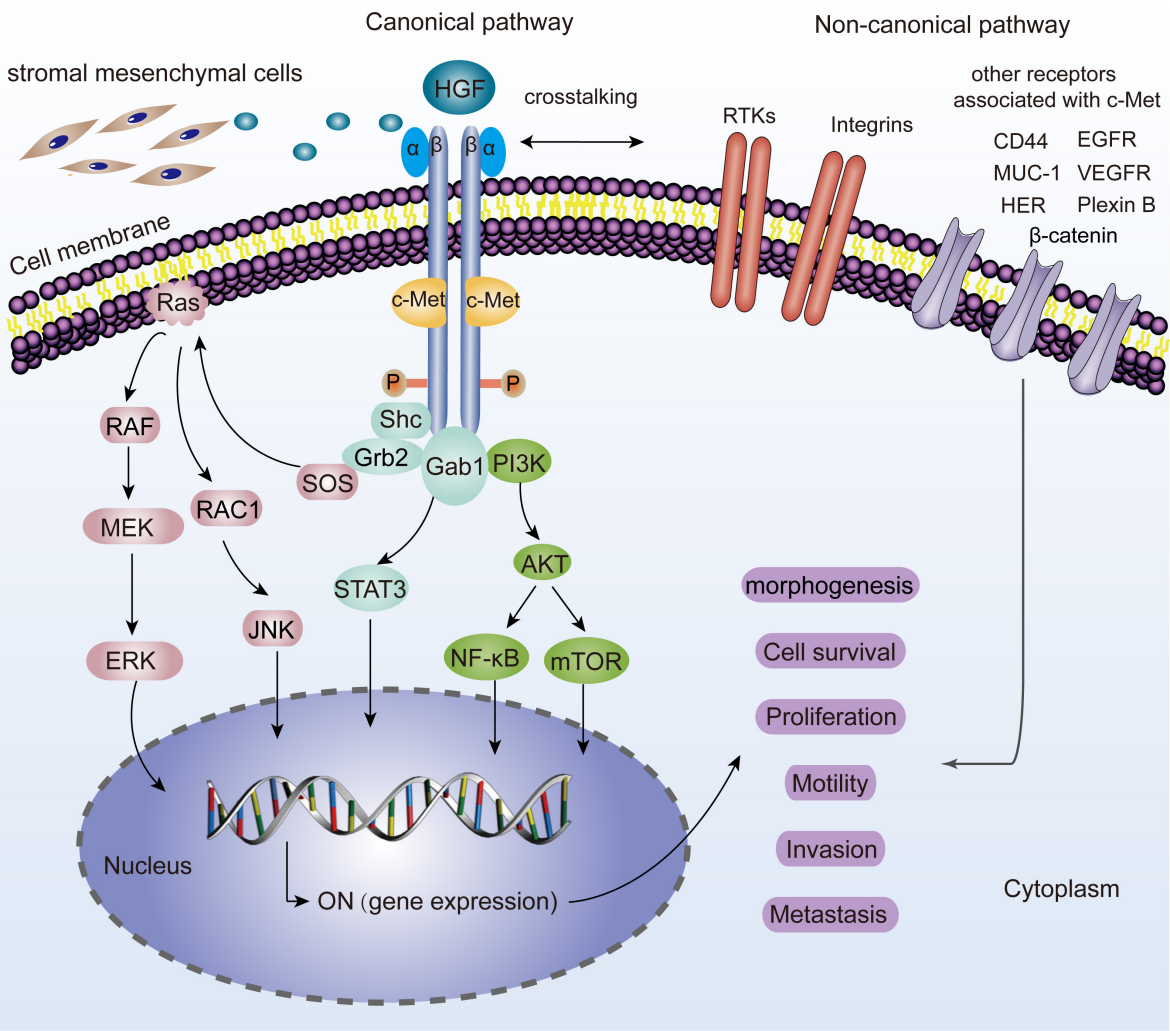
Representation of the HGF/c-Met canonical and non-canonical pathways. For canonical pathways, the binding of HGF, induces two c-Met molecules dimerization, thereby leading to the autophosphorylation of tyrosine residues and subsequent activation of many downstream signaling pathways such as MAPK/ERK, STAT3, PI3K/AKT signaling. JNK is also phosphorylated and activates a variety of downstream substrates, including transcription factors such as AP-1 and apoptosis-related Bcl-2, Bax, etc. All these hereby basically drive a plethora of cell phenotypes such as morphogenesis, survival, proliferation, motility, invasion, and metastasis. “ON” means the activation for gene expression. Non-canonical pathways are activated when c-Met binds to other receptors including EGFR, MUC-1, VEGFR, CD44, Plexin B1, HER, Integrin α6β4, β-catenin, and so on.

C-Met is located on chromosome 7q21-31 and belongs to the family of receptor tyrosine kinases (Zhang et al., 2018). It is a heterodimer composed of a highly glycosylated extracellular 50 kDa α-chain and a transmembrane 140 kDa β-chain, linked together by disulfide bonds (Baldanzi and Graziani, 2014). As a natural HGF receptor, it is expressed on the surfaces of various epithelial cells and is known for its roles in promoting tumorigenesis. After binding to the HGF, the two subunits are dimerized, leading to the autophosphorylation of two tyrosine residues (Tyr1234 and 1235). The autophosphorylation of the tyrosine residues results in an activated multifunctional docking site composed of Src homology-2 (SH2) domain, phosphotyrosine binding (PTB) domain, and Met binding domain (MDB), which recruit adapter molecules (Furge et al., 2000). For example, Grb-2, which is a vital element in HGF/c-Met axis, interacts with Y1356 of c-Met and several essential signaling pathway-related proteins such as Ras, SOS, and Gab1 and eventually activates them. Besides, these residues also recruit PI3K, STAT3, PLCγ, and SHP2 thereby linking oncogenes and many receptors which are crucial for cellular functions (Garcia-Vilas and Medina, 2018). Moreover, it has been revealed that c-Met could be activated by non-canonical pathways. Non-canonical pathways are related to c-Met amplification, drug-resistant cancers and malignant tumor features. Several membrane surface proteins (EGFR, β-catenin, MUC-1, CD44, VEGFA, Plexin B, FAK, HER, Integrin α6β4, Fas, etc.) have been confirmed to interact with c-Met and contribute to dynamic c-Met biological responses (Migliore and Giordano, 2008; Scagliotti et al., 2013). The molecular network of the HGF/c-Met signaling is shown in Figure 1.

## Crosstalk Between HGF/c-Met Axis and ncRNAs in Cancers

**Figure 2:**
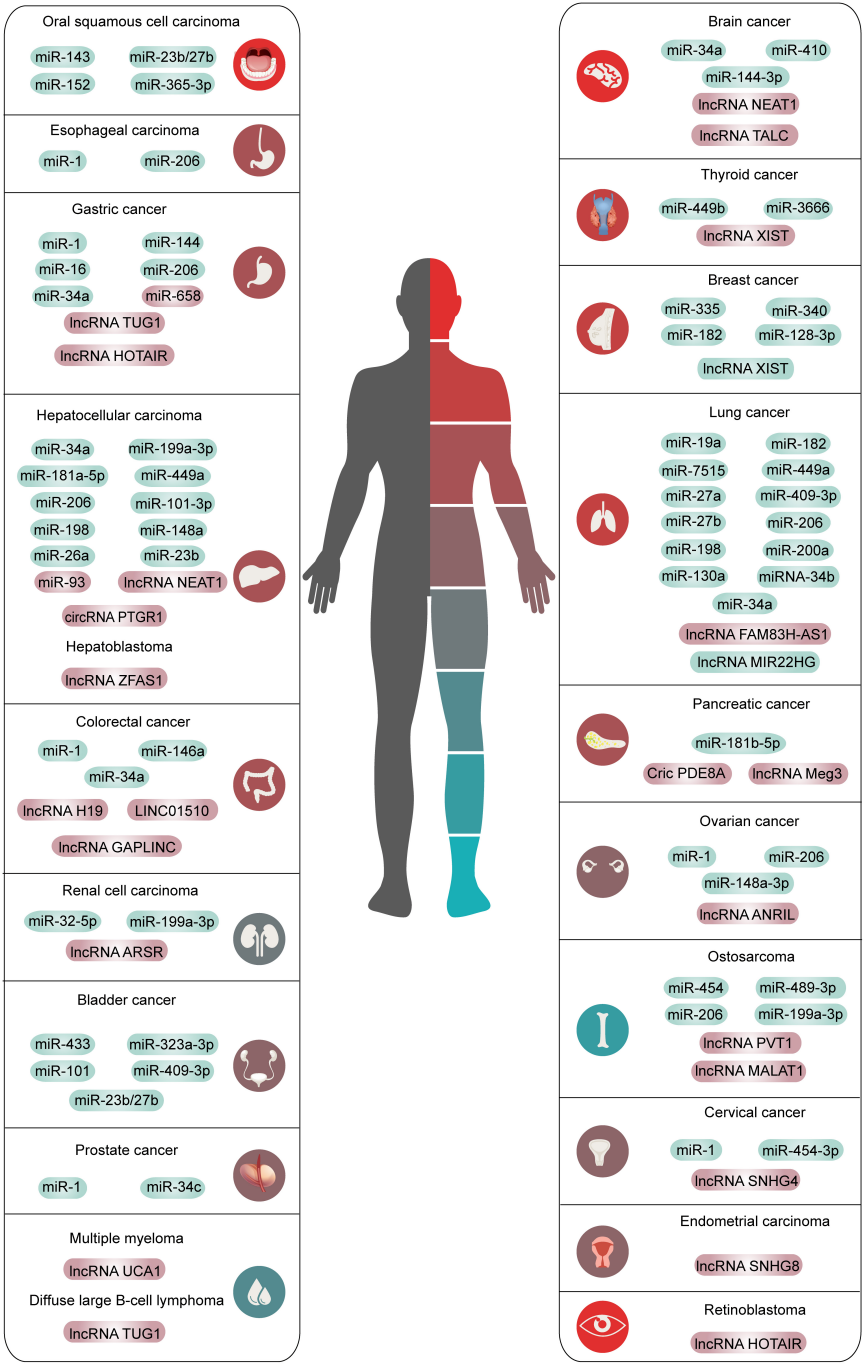
The overview of ncRNAs interacted with HGF/c-Met axis in human common malignancies. The red means high expression and the green means low expression.

This chapter summarizes the interaction between the HGF/c-Met axis and ncRNAs in common cancers. The outline is shown in Figure 2, Tables 1, 2.

**Table 1:** The crosstalk between microRNAs and HGF/c-Met axis in cancers.

**NcRNAs**	**Year**	**Expression status in cancers**	**Role in cancers**	**Mechanism**
**LUNG CANCER**				
MiR-7515	2013	Downexpression	Inhibit proliferation and migration	Targeting c-Met and altering the signaling of downstream cell-cycle-related proteins
MiR-27a	2013	/	/	Targeting MET and EGFR
MiR-27b	2017	Downexpression	Inhibit the proliferation, migration, and invasion	Targeting Met
MiR-34a	2015	Downexpression	Serve as a prognostic factor for recurrence	Regulating c-Met and CDK6 expression
MiR-34b	2013	Downexpression	Induce cell apoptosis	Regulating phospho-Met, P53 and Mdm2 expression
MiR-409-3p	2014	Downexpression	Inhibit growth, induce apoptosis, reduce migration, and invasion	Regulating Akt signaling pathway by targeting c-Met
MiR-449a	2013	Downexpression	Inhibit migration and invasion	Targeting c-Met
MiR-182	2018	Downexpression	Inhibit HGF-induced migration, invasion, and EMT	Regulating c-Met/AKT/Snail signaling pathway
MiR-206	2015, 2016	Downexpression	Inhibit the proliferation, migration, invasion, angiogenesis and induced apoptosis, as well as increase c-Met induced cisplatin resistance	Targeting MET, BCL2, and c-Met/PI3k/Akt/mTOR pathway
MiR-130a	2014	Downexpression in gefitinib resistant NSCLC cell lines	Inhibit the resistance of NSCLC cells to gefitinib	Downregulation of Met by directly targeting its 3′-UTR
MiR-19a	2017	Downexpression in gefitinib-resistant NSCLC cell lines	contribute to cell migration and EMT	Targeting c-Met and regulating its downstream pathway such as AKT and ERK pathways
MiR-198	2018	Downexpression	Inhibit proliferation, migration, and invasion, induces apoptosis, and overcome resistance to radiotherapy	Targeting HGF/c-MET signaling pathway
MiR-200a	2015, 2018	Downexpression	Inhibit migration, invasion, gefitinib resistance, and enhance the radiosensitivity of NSCLC cells	Targeting EGFR and c-Met, and regulating HGF/c-Met signaling pathway
**HEPATOCELLULAR CARCINOMA**				
MiR-34a	2009, 2013	Downexpression	Inhibit migration and invasion	Directly targeting c-Met
MiR-206	2019	Downexpression	Inhibit cell proliferation and migration but promote apoptosis	Regulating c-Met
MiR-23b	2009	Downexpression	Reduce the migration and proliferation abilities	Mediating urokinase and c-Met
MiR-199a-3p	2010	Downexpression	Influence the doxorubicin sensitivity of human hepatocarcinoma cells; suppress tumor growth, migration, invasion and angiogenesis	Regulating mTOR and c-Met; Targeting VEGFA, VEGFR1, VEGFR2, HGF and MMP2
MiR-198	2011	Downexpression	Inhibit migration and invasion	Targeting HGF/c-Met pathway
MiR-148a	2014	Downexpression	Suppress the EMT and metastasis	Targeting Met/Snail signaling
MiR-181a-5p	2014	Downexpression	Suppress motility, invasion, and branching-morphogenesis	Directly targeting c-Met
MiR-449a	2018	Downexpression	Suppress hepatocellular carcinoma cell growth	Regulating CDK6 and c-Met/Ras/Raf/ERK signaling
MiR-101-3p	2019	Downexpression	Suppress proliferation and migration	Targeting HGF/c-Met pathway
MiR-93	2015	Overexpression	Increase HCC cells proliferation, migration, invasion, and the resistance to sorafenib and tivantinib treatment	Activating c-Met/PI3K/Akt pathway
MiR-26a	2014	Downexpression	Suppress angiogenesis	Targeting HGF/c-Met pathway
**GASTRIC CANCER**				
MiR-1	2015	Downexpression	Inhibit cell proliferation and migration	Targeting MET
MiR-144	2015	Downexpression	Inhibit metastasis and proliferation	Directly binding the 3′-UTR of MET mRNA
MiR-16	2016	Downexpression	Inhibit proliferation and migration	Directly targeting 3′- UTR of HGF mRNA
MiR-658	2018	Overexpression in metastatic gastric group	Promote migration and invasion	Not explored. But its expression was positively associated with PAX3 and MET
MiR-206	2015	Downexpression	Suppress proliferation, migration and invasion *in vitro* and *in vivo*, meanwhile induce G1 phase arrest	Targeting c-Met; Through miR-206/PAX3/MET axis
MiR-34a	2014, 2015, 2016	Downexpression, particularly in DDP resistance patients and cells	Inhibit the growth, invasion, and metastasis, meanwhile increase the sensitivity to DDP	Regulating PDGFR and MET expression through PI3K/Akt/mTOR pathway
**OSTEOSARCOMA**				
MiR-199a-3p	2011	Downexpression	Inhibit proliferation, migration, and induce G1 phase arrest	Not explored, but might associated with mTOR, c-Met and Stat3
MiR-454	2015	Downexpression	Suppress cell proliferation and invasion	Directly targeting c-Met
MiR-489-3p	2017	Downexpression	Suppress proliferation and metastasis *in vitro* and *in vivo*	Regulating PAX3-MET axis
MiR-206	2019	Downexpression	Suppress cell proliferation and metastasis, as well as increase cell apoptosis	Targeting PAX3 and MET
**BREAST CANCER**				
MiR-335	2014	Downexpression	Suppress breast cancer cell migration	Targeting c-Met
MiR-340	2011	Downexpression	Suppress cell migration and invasion	Targeting c-Met
MiR-128-3p	2018	Downexpression	Suppress cell migration and invasion	Targeting c-Met
MiR-182	2019	Downexpression in trastuzumab resistant cells	Reduce invasion and migration and induce the impediment to trastuzumab resistance	Through MET-dependent PI3K/Akt/mTOR pathway
**BLADDER CANCER**				
MiR-23b/27b	2014	Downexpression	Inhibit cell proliferation, migration, and invasion	via the attenuation of EGFR and c-Met
MiR-101	2013	Downexpression	Suppress motility of bladder cancer cells	Targeting c-Met
MiR-323a-3p	2017	Downexpression	Suppress EMT progression	Through mMET/SMAD3/SNAIL circuit
MiR-409-3p	2013	Downexpression	Inhibit migration and invasion	Directly targeting c-Met and indirectly regulating MMP2 and MMP9
MiR-433	2016	Downexpression	Suppress cell proliferation, migration and invasion	Directly targeting CREB1 and c-Met, and regulating Akt/GSK-3β/Snail signaling
**COLORECTAL CANCER**				
MiR-1	2012	Downexpression	Impair MET-induced proliferation, migration, and invasion programs	Inversely regulating MET
MiR-34a	2013	Downexpression	Predict distant metastasis of colon cancer	Might associate with its targets c-Met, Snail and β-catenin
MiR-146a	2018	Downexpression	Abolish colorectal cancer liver metastasis	Targeting c-Met
**GLIOMA**				
MiR-410	2012	Downexpression	Attenuate tumor growth and invasion	Inhibiting MET and AKT signaling
MiR-34a	2009	Downexpression	Inhibit cell proliferation, cell cycle progression, cell survival, and cell invasion	Targeting c-Met, Notch-1, and Notch-2
MiR-144-3p	2015	Downexpression	Inhibit proliferation and invasion	Binding to c-Met
**OVARIAN CANCER**				
MiR-1	2017	Downexpression	Compromise proliferation, survival, invasion, and metastasis	Directly targeting c-Met
MiR-206	2018	Downexpression	Compromise proliferation, survival, invasion, and metastasis	Directly targeting c-Met
MiR-148a-3p	2018	Downexpression	Compromise proliferation, survival, invasion, and metastasis	Directly targeting c-Met
**CERVICAL CANCER**				
MiR-454-3p	2018	Downexpression	Impede proliferation, migration, and invasion	Targeting c-Met
MiR-1	2019	Downexpression	Trigger the proliferation, migration, and infiltration of cancer cells	Be associated with c-Met
**PANCREATIC DUCTAL ADENOCARCINOMA (PDAC)**				
MiR-181b-5p	2017	Downexpression in radiation-resistant PDAC cells	/	Through miR-181b-5p/ETS1/c-Met axis
**PROSTATE CANCER**				
MiR-34c	2013	Downexpression	Suppress migration	Targeting MET
MiR-1	2019	Downexpression	Suppress migration and proliferation	Regulating c-Met/PI3K/Akt/mTOR signaling
**ORAL SQUAMOUS CELL CARCINOMA (OSCC)**				
MiR-143	2015	Downexpression	Dampen OSCC cell mobility	Downregulating of phospho-c-Met through targeting CD44 v3
MiR-23b/27b	2016	Downexpression	Inhibit proliferation, migration, and invasion	Targeting MET
MiR-152	2018	Downexpression	Inhibit proliferation, migration, invasion	Targeting c-Met
MiR-365-3p	2019	Downexpression	Inhibit OSCC migration, invasion, metastasis and chemoresistance to 5-fluorouracil	Targeting miR-365-3p/EHF/KRT16/β5-integrin/c-met signaling axis
**RENAL CELL CARCINOMA**				
MiR-199a-3p	2014	Downexpression	Inhibit proliferation and caused G1 phase arrest	Suppressing HGF/c-Met axis and its downstream signaling
MiR-32-5p	2018	Downexpression	Suppress metastasis and enhance the efficacy of sunitinib-chemotherapy	Regulating TR4/HGF/Met axis
**THYROID CANCER**				
MiR-449b	2015	Downexpression	Inhibit tumor proliferation	Targeting MET
MiR-3666	2016	Downexpression	Inhibit tumor proliferation	Targeting MET
**ESOPHAGEAL SQUAMOUS CELL CARCINOMA (ESCC)**				
MiR-1	2016	Downexpression	Inhibit cell growth and enhance of cell apoptosis	Downregulating of MET, cyclin D1, and CDK4 expression
MiR-206	2019	Downexpression	Inhibit cell proliferation and induced apoptosis	Regulating c-Met/AKT/mTOR axis

**Table 2:** The crosstalk between lncRNAs and cricRNAs and HGF/c-Met axis in cancers.

**NcRNAs**	**Year**	**Expression status in cancers**	**Role in cancers**	**Mechanism**
**LUNG CANCER**				
LncRNA FAM83H-AS1	2017	Overexpression	Increase cell proliferation, invasion, and migration	Targeting MET/EGFR signaling pathway
lncRNA MIR22HG	2018	Downexpression	Inhibit cell proliferation, colony formation, migration, and invasion	Through regulating YBX1, MET and P21 expression
**HEPATOCELLULAR CARCINOMA**				
LncRNA NEAT1	2019	Overexpression	Suppress sorafenib sensitivity	Regulating miR-335–c-Met axis
CircRNA PTGR1	2019	Overexpression in highly metastatic cells	Enhance the metastatic potential of lower metastatic cells	Regulating miR-449a/MET interaction
**GASTRIC CANCER**				
LncRNA HOTAIR	2015	Overexpression	Promote migration, invasion, and metastasis	Through lncRNA HOTAIR/miR-34a/HGF/Met/Snail pathway by binding to PRC2
LncRNA TUG1	2016	Overexpression	Promote transference and invasion	Through lncRNA TUG1/miR-144/c-Met axis
**OSTEOSARCOMA**				
LncRNA MALAT1	2019	Overexpression	Promote proliferation, migration, and invasion of OS cells *in vitro*	Targeting c-Met and SOX4 via miR-34a/c-5p and miR-449a/b
LncRNA PVT1	2019	Overexpression	Enhance chemoresistance of gemcitabine	Through miR-152/c-MET/PI3K/AKT pathway
**BREAST CANCER**				
lncRNA XIST	2018	Downexpression in brain metastasis tissue	Inhibit brain metastasis and EMT and stemness process	By activating c-Met pathway via upregulating MSN and reprogramming microglia via secreting exosomal miR-503
**COLORECTAL CANCER**				
LncRNA GAPLINC	2018	Overexpression	Stimulate cells migration and invasion	Regulating miR-34a/c-MET Signal Pathway
LncRNA 01510	2018	Overexpression	Promote proliferation and cell cycle arrest in G1 phase	Regulating MET
LncRNA H19	2019	Overexpression	/	Positive correlated with MET expression
**GLIOMA**				
LncRNA NEAT1	2015	Overexpression	Promote cell proliferation, migration, and invasion and inhibit apoptosis	Regulating miR-449b-5p/c-Met axis
**GLIOBLASTOMA (GBM)**				
LncRNA TALC	2019	Overexpression in TMZ-resistant cells	Promote TMZ resistance	By trapping miR-20b-3p, activating c-Met and increasing MGMT expression
**SEROUS OVARIAN CANCER (SOC)**				
LncRNA ANRIL	2015	Overexpression	Promote cell migration and invasion	Regulating MET and MMP3
**CERVICAL CANCER**				
LncRNA SNHG4	2019	Overexpression	Promote cell proliferation and inhibit apoptosis	Regulating miR-148a-3p /c-Met axis
**ENDOMETRIAL CANCER**				
LncRNA SNHG8	2018	Overexpression	Promote cell proliferation	Regulating miR-152/c-MET axis
**PANCREATIC CANCER**				
Circ-PDE8A	2018	Overexpression	Stimulate tumor migration and growth	Through miR-338/MACC1/MET/AKT or ERK pathway
LncRNA Meg3	2015	Downexpression	Block cell growth and delay cell cycle progression	Negatively regulating c-Met
**RENAL CELL CARCINOMA**				
LncRNA ARSR	2016	Overexpression	Promote sunitinib resistance	Through via competitively binding miR-34 and miR-449 to promote AXL and c-MET expression
LncRNA NEAT1	2017	Overexpression	Enhance EMT and chemoresistance to sorafenib	Via the miR-34a/c-Met axis
**THYROID CANCER**				
LncRNA XIST	2018	Overexpression	Inhibit the cell proliferation and tumor growth	Via LncRNA XIST/miR-34a/MET/PI3K/AKT axis
**MULTIPLE MYELOMA (MM)**				
LncRNA UCA1	2019	Overexpression	Facilitate proliferation and reduce apoptosis	Through lncRNA UCA1/miR-1271-5p/HGF axis
**HEPATOBLASTOMA (HB)**				
LncRNA ZFAS1	2019	Overexpression	Promote proliferation and invasion	Through lncRNA ZFAS1/ miR-193a-3p/RALY/HGF/c-Met pathway
**RETINOBLASTOMA**				
LncRNA HOTAIR	2018	Overexpression	Promote proliferation and EMT process but impair apoptosis	Through miR-613/c-met axis
**DIFFUSE LARGE B-CELL LYMPHOMA (DLBCL)**				
LncRNA TUG1	2019	Overexpression	Facilitate proliferation and reduce apoptosis	Through increasing MET expression by inhibiting its ubiquitination and degradation

### Lung Cancer

Lung cancer is one of the most frequent cancers globally. In 2018, it contributed to 11.6% of all the new cancer cases worldwide (Bray et al., 2018). The disease can be classified into two primary subtypes: non-small cell lung cancer (NSCLC) and small-cell lung cancer, representing 85 and 15% of all lung cancer cases, separately. Lung cancer is very aggressive, more than 50% of the patients die within 1 year after diagnosis, and the 5-year survival rate is below 18% (Sun R. C. et al., 2019). It is still difficult to disclose the indisposition at an early primary phase for possible therapeutic and surgical treatments.

Lee et al. were the first to identify and characterize a novel miR-7515 from lung cancer cells. The miRNA has been shown to inhibit tumor proliferation and migration by directly repressing c-Met expression and altering the signaling of downstream cell cycle-related proteins (Lee et al., 2013). Subsequently, several miRNAs have been reported to regulate c-Met in NSCLC. For example, studies demonstrated that suppression of miR-27a (Acunzo et al., 2013), miR-27b (Zhou et al., 2017), miR-34a (Hong et al., 2015), and miR-34b (Wang et al., 2013) impairs NSCLC progression by regulating c-Met. Another tumor suppressor, miR-409-3p, negatively correlates with c-Met and has been shown to undermine tumor growth, migration, and invasion. Also, it induced apoptosis *in vitro* via the Akt signaling pathway by targeting c-Met (Wan et al., 2014). Luo et al. revealed that upregulation of miR-449a was significantly associated with less lymph node metastasis and better prognosis in NSCLC via regulation of the target gene c-Met (Luo et al., 2013). Consistently, miR-182 was found to inhibit HGF-induced migration, invasion, and EMT by regulating c-Met/AKT/Snail signaling pathway in NSCLC (Li Y. et al., 2018). Furthermore, Chen et al. showed that miR-206 could inhibit HGF-induced EMT and angiogenesis and induce cisplatin resistance by regulating c-Met/PI3K/Akt/mTOR pathway in lung cancer (Chen Q. Y. et al., 2015; Chen et al., 2016a,b).

The HGF/c-Met axis also contributes to drug resistance in lung cancer. Zhou et al. were the first to verify that miR-130a is highly expressed in gefitinib-sensitive NSCLC cell lines, but not gefitinib-resistant NSCLC cells. They also demonstrated that elevated miR-130a could enhance the sensitivity of NSCLC cells to gefitinib and promote apoptosis by directly reducing c-Met levels (Zhou et al., 2014). Similarly, decreased miR-19a expression in gefitinib-resistant NSCLC cell lines contributes to cell migration, EMT, and gefitinib resistance by negatively regulating c-Met expression and blocking its downstream pathways (such as PI3K-AKT and RAS-ERK pathways) (Cao et al., 2017). Zhu et al. demonstrated that miR-198 could inhibit proliferation, migration, and invasion, induce apoptosis, and overcome resistance to radiotherapy via HGF/c-Met axis. It was downregulated in NSCLC cell lines but overexpressed in radiotherapy sensitive patients (Zhu et al., 2018). Studies also reported that miR-200a plays an active tumor-suppressing role in non-small cell lung cancer, and could also inhibit gefitinib resistance and enhance the radio-sensitivity by deactivating the HGF/c-Met pathway (Zhen et al., 2015; Du et al., 2019).

Moreover, lncRNA FAM83H-AS1 was suggested as a potential therapeutic target that could accelerate cell proliferation, invasion, and migration by activating MET/EGFR signaling pathway in lung adenocarcinoma (Zhang et al., 2017). Su et al. also identified the anticancer effect of lncRNA MIR22HG and there was a significant association between lncRNA MIR22HG and MET expression (Su et al., 2018). These studies provided new insights into prognostic diagnosis and therapeutic strategies for patients with lung cancer. Collectively, the HGF/c-Met axis and ncRNAs play an essential role in the formation and development of lung cancer.

### Hepatocellular Carcinoma (HCC)

HCC is one of the ordinary cancers with the highest morbidity and mortality globally. Till now, the underlying molecular mechanisms for pathogenesis and development of HCC remain mostly unknown because of multiple etiologies (Bray et al., 2018). Therefore, studies on the molecular checkpoints involved in HCC development and aggressiveness are crucial.

In 2009, Li et al. were the first to demonstrate that miR-34a is less expressed in HCC tissues than in corresponding normal tissues. In the same study, it was revealed that miR-34a HCC reduces malignant phenotype by downregulating c-Met expression and decreasing the phosphorylation level of ERK1/2 (Li N. et al., 2009). Later, miR-206 (Wang Y. et al., 2019) and miR-23b (Salvi et al., 2009) were found to attenuate cell viability, migration, and invasion by blocking the c-Met activation in HCC. The study concluded that miR-199a-3p is closely associated with the formation and progression of HCCs. In the same study, a significant increase in the G1-phase population following the restoration of miR-199a-3p expression was observed. Further investigation of the mechanism showed that miR-199a-3p contributed to the cell cycle modulation by silencing both mTOR and c-Met (Fornari et al., 2010; Ghosh et al., 2017). MiR-198 bound to the 3′ UTR of c-Met mRNA and blocked p44/42 MAPK activity through HGF/c-Met pathway, leading to the inhibition to migration and invasion of HCC cells (Tan et al., 2011). Consistently, miR-148a was reported to impede EMT and metastasis by targeting HGF/Met/Snail signaling in HCC (Zhang et al., 2014). Besides invasion, miR-181-5p suppressed branching-morphogenesis by directly regulating c-Met (Korhan et al., 2014). Many studies have shown that miR-449a impairs the EMT of tumors by targeting MET, but the precise single target of miR-449a has not been elucidated in HCC (Sun et al., 2017). Cheng et al. revealed that miR-449a suppressed HCC growth by targeting CDK6 and impairing c-Met/Ras/Raf/ERK signaling pathway with an iTRAQ proteomics approach. Their study linked miR-449a, cell cycle, and c-Met/Ras/Raf/ERK signaling pathway in HCC and could also explain the abnormal growth characteristics of HCC cells (Cheng et al., 2018). Besides, *in vitro* and *in vivo* studies have demonstrated that miR-101-3p inhibits HCC progression by targeting HGF and it could be regarded as another potential therapeutic tool for regulating cell proliferation and metastasis (Liu et al., 2019). However, miR-93 was identified as HCC stimulator, which increased HCC cell proliferation, migration, and invasion by activating c-Met/PI3K/Akt pathway (Ohta et al., 2015). Thus, HGF/c-Met axis might be of high interest in regulating tumor progression in HCC.

In our review, the potential roles of the HGF/c-Met axis in other hallmarks of HCC, such as interactions with tumor microenvironment and drug resistance, are also highlighted. VEGFA/VEGFR2 signaling is a crucial downstream pathway of HGF/c-Met axis and plays a vital role in tumor angiogenesis. VEGFA secreted from cancer cells binds to VEGFR2 on endothelial cells, phosphorylates and activates VEGFR2, which then phosphorylates downstream extracellular signal-regulated kinase (ERK1/2) thus promoting angiogenesis (Ferracini et al., 1995; Fischer et al., 2004; Testini et al., 2019). Recently, Yang et al. confirmed that miR-26a could inhibit tumor proliferation and metastasis of HCC through IL-6-Stat3 signaling pathway. Notably, a negative correlation between miR-26a and VEGFA or microvessel density (MVD), two well-known proangiogenic factors, was observed. MiR-26a significantly impaired *in vivo* tumor angiogenesis by inhibiting VEGFA production through HGF/c-Met axis in HCC cells. Moreover, miR-26a also impaired VEGFR2 signaling in endothelial cells to exert its antiangiogenesis function (Yang et al., 2014). Accordingly, miR-199a-3p showed important inhibitory effect on angiogenesis by targeting HGF and subsequently blocking downstream signaling pathways. Ghosh et al. demonstrated that miR-199a-3p reduced VEGF secretion in HCC cells and inhibited expression of VEGFR1 and VEGFR2 receptors on endothelial cells. Besides, MMP2 signaling was abrogated by miR-199a-3p (Ghosh et al., 2017). Furthermore, HGF/c-Met axis was reportedly involved in drug resistance. MiR-199a-3p could regulate the doxorubicin sensitivity of human HCC cells through c-Met and mTOR (Fornari et al., 2010). Katsuya revealed that overexpression of miR-93 could improve the resistance of HCC cells against sorafenib and tivantinib treatment through c-Met/PI3K/Akt pathway (Ohta et al., 2015).

With regards to lncRNAs, Chen et al. demonstrated that lncRNA NEAT1 could suppress the sorafenib sensitivity of HCC cells by regulating miR-335/c-Met pathway (Chen and Xia, 2019). In addition, exosomes derived from highly metastatic cells (LM3) with a high abundance of circPTGR1 might enhance the metastatic potential of lower metastatic cells (97L and HepG2) by inhibiting miR-449a-MET interaction, resulting in destruction on tumor microenvironment and thereby promoting HCC development (Wang G. et al., 2019). Collectively, the results of these studies show that HGF/c-Met axis is an effective multifaceted tumor regulator, which could have pivotal implications on the understanding of HCC mechanisms and the improvement of therapeutics.

### Gastric Cancer (GC)

GC is one of the most ordinary neoplasms with a high incidence, especially in Eastern Asia. Metastasis is the main reason for its which results in high mortality of the patients (Bray et al., 2018). Nonetheless, the precise molecular mechanisms underlying GC metastasis have not uncovered.

The HGF/c-Met axis has been implicated as a crucial modulator of metastasis-associated signal transduction pathways in GC progression. Several miRNAs have been reported to participate in the GC proliferation and metastasis by directly or indirectly regulating the expression of c-Met. MiR-1 (Han et al., 2015) and miR-144 (Liu J. et al., 2015) are downregulated in GC tissues and directly target the 3′-UTR of MET mRNA, thus, leading to the repression of GC progression. Besides, downregulation of miR-16 suppresses HGF expression by binding to its 3′-UTR (Li et al., 2016). In a previous study, the expression of miR-658 was elevated in distant GC cells, which increased the levels of serum miR-658 significantly, thus promoting cell migration and invasion. Besides, a strong positive relation between serum level of miR-658 and mRNA PAX3 and MET expression has been observed (Wu et al., 2018). However, the precise role of miR-658/PAX3/MET axis needs further exploration.

MiR-206, a famous tumor suppressor, is thought to enhance tumor metastasis by regulating diverse mRNA expression at a post-transcription level in some forms of cancer such as breast and colon cancers. In a study by Zhang et al., the expression of miR-206 was weaker in GC cell lines, especially in high metastatic cell lines. Transwell assay confirmed that the ectopic expression of miR-206 inhibited the migration and invasion ability of GC cells by regulating PAX3-MET pathways. *In vivo* mouse experiments demonstrated that miR-206 overexpression resulted in significant decrease in the incidence of lung metastasis and the number of metastatic lung nodules (Zhang et al., 2015). The study from Zheng et al. also supported the conclusion that miR-206/MET axis modulates the migration and invasion of GC. Besides, suppression of tumor growth was observed after miR-206 mimics transfection, which indicated downregulation of c-Met and cell cycle-related proteins in xenograft mouse models and *in vitro* (Zheng et al., 2015).

MiR-34a also has tumor suppression ability. A study reported that miR-34a, which was weakly expressed in GC tissues, attenuated the malignant behavior of GC cells by directly targeting PDGFR and MET expression and subsequently regulating the phosphorylation of Akt through PI3K/Akt/mTOR pathway (Peng et al., 2014; Wei et al., 2015). In addition to these findings, Zhang et al. revealed that miR-34a could modulate human GC cells cisplatin (DDP) sensitivity by regulating cell proliferation and apoptosis by targeting MET. Specifically, they found that miR-34a expression was significantly less in DDP resistance human GC tissues and cells than in normal GC tissues and cells. The overexpression of miR-34a enhanced the DDP sensitivity of SGC7901/DDP cells by inhibiting cell proliferation and inducing cell apoptosis. Therefore, its downregulation could weaken the sensitivity to DDP. A subsequent study confirmed that miR-34a modulates DDP resistance by directly repressing MET (Zhang et al., 2016). Collectively, miR-34a could contribute to the development of new therapeutic strategies for gastric cancer.

Furthermore, competing endogenous RNAs (ceRNAs) as essential miRNA-regulators have emerged as therapeutics scavenging oncogenic miRNAs. Recently, they have exposed exciting insights into tumorigenesis. LncRNA HOTAIR was found to be overexpression in GC tissue and cells, particularly in diffuse-type GC. In addition, lncRNA HOTAIR knockdown remarkably impaired migration, invasion and metastasis both *in vitro* and *in vivo* and reversed the EMT in GC cells by epigenetically inhibiting miR34a through recruiting and binding to PRC2, mechanically (Liu Y. W. et al., 2015). Similarly, Ji et al. elucidated that overexpression of lncRNA-TUG1 led to a significant proliferation of GC tissue (most likely) by inhibiting miR-144. Also, lncRNA-TUG1 indirectly activated the expression of c-Met, thus promoting the metastasis of GC cells via lncRNA-TUG1/miR-144/c-Met axis (Ji et al., 2016).

### Osteosarcoma (OS)

OS predominantly originates from mesenchymal cells of long bones and has a high prevalence in children and young adults. It usually starts as a monoclonal disease and quickly progresses into a polyclonal disease. It is regarded as one of the most complicated cancers in terms of molecular aberration (Kansara and Thomson, 2019). Further elucidation of its exact molecular mechanism could be useful in the identification of its associated markers, which might assist in prognosis and therefore improve its treatment.

In 2011, Duan et al. were the first to reveal the negative association between the expression of miR-199a-3p and mTOR, c-Met and Stat3 (Duan et al., 2011). But the molecular mechanism behind the tumor suppressor role of miR-199a-3p in human OS has not been fully elucidated. MiR-454 was found to be downregulated in OS clinical samples and cell lines. And this suppressed proliferation and metastasis of OS cells by inhibiting c-Met expression (Niu et al., 2015). Studies conducted by Liu et al. suggested that miR-489-3p expression could inhibit the proliferation and metastasis of OS tissues and cells, particularly in high metastatic potential cells by negative regulation of PAX3-MET axis. Also, xenografts study results showed smaller cancer nodules and fewer incidences of lung metastases in LV-miR-489-3p group compared with LV-control group, indicating the apparent inhibition of OS metastasis *in vivo* (Liu Q. et al., 2017). Similarly, miR-206 was reported to repress OS progression by targeting PAX3-MET axis (Zhan et al., 2019).

ceRNAs also play critical roles in the development of OS. Sun et al. demonstrated that serum level of long non-coding RNA Metastasis-Associated Lung Adenocarcinoma Transcript 1 (lncRNA MALAT1) was higher in metastatic OS tissues than non-metastasis OS tissues or corresponding normal tissues. In the same study, elevated levels of serum lncRNA MALAT1 predicted worse clinical outcomes in OS patients. The silencing of lncRNA MALAT1 was shown to inhibit proliferation, migration, and invasion of OS cells. Bioinformatic and molecular analyses revealed that lncRNA MALAT1 served as a ceRNA of c-Met and SOX4 by directly targeting miR-34a/c-5p and miR-449a/b (Sun Z. et al., 2019). The function of ceRNAs in drug resistance has also been elucidated. LncRNA PVT1 played critical roles in chemoresistance of OS to gemcitabine (GEM) and was upregulated in OS drug-resistant cells. Functional studies have demonstrated that lncRNA PVT1 could reduce GEM-reduced apoptosis and attenuate GEM-induced tumor growth inhibition *in vitro* and *in vivo*. A study on lncRNA PVT1 mechanism revealed that lncRNA PVT1 targeted miR-152, which promoted chemoresistance of OS by activating c-MET/PI3K/AKT pathway (Sun Z. Y. et al., 2019). These findings revealed a novel interaction network that could eventually lead to new therapeutic strategies for OS patients based on ncRNAs.

### Breast Cancer

Breast cancer is the leading cause of mortality in women worldwide (Colditz and Bohlke, 2014). It occurs as a consequence of various signaling pathways in mammary epithelial cells, including the HGF/c-Met axis. Many studies have shown that the interaction between ncRNAs and HGF/c-Met axis exerts regulatory effects on breast cancer progression and clinical therapy. Recent studies have suggested that miR-335 could be upregulated in breast cancer tissues. Overexpression of miR-335 has been shown to suppress HGF-induced phosphorylation of c-Met, which could subsequently inhibit the HGF role of prompting breast cancer cell migration in a c-Met-dependent manner by targeting c-Met (Gao et al., 2015). Wu and Breunig confirmed the hypothesis of post-transcriptional regulation of MET by revealing that miR-340 and miR-128-3p negatively regulate MET expression, thus inhibiting the invasion and migration of breast cancer cells (Wu et al., 2011; Breunig et al., 2018).

Concerning drug resistance, Yue et al. revealed that miR-182 binds to MET in breast cancer cells and suppresses the cell's ability to tolerate trastuzumab, therefore reducing the invasion and migration capability of trastuzumab-resistant cells through MET-dependent PI3K/AKT/mTOR signaling pathways (Yue and Qin, 2019). This study highlighted the importance of the interaction between ncRNAs and HGF/c-Met axis in cancer development and therapy.

For lncRNAs, in 2018, Xing et al. profiled lncRNAs in breast cancer tissue with brain metastasis and confirmed that the X-inactive–specific transcript (XIST) was remarkably downregulated. Further *in vitro* and *in vivo* experiments revealed that knockdown lncRNA XIST could activate c-Met pathway via upregulating MSN to promote EMT and stemness. Meanwhile, lncRNA XIST^low^ cells could reprogram microglia in the brain via secreting exosomal miR-503. And the prometastatic effect was inhibited by fludarabine (Xing et al., 2018). These indicated that lncRNA XIST might become an effective target for curing brain metastasis.

### Bladder Cancer (BCa)

BCa is the most frequent cancer of the urinary tract (Grayson, 2017). Chlyomaru et al. were the first to reveal the function of miRNA-23b/27b cluster in bladder cancer. In their study, the capacities of cell proliferation, migration, and invasion of bladder cancer cells were repressed after restoring miR-23b and miR-27b expression by attenuation of EGFR and c-Met (Chiyomaru et al., 2015). Hu et al. reported a similar mechanism in miR-101 (Hu et al., 2013). MiR-323a-3p is a member of the miRNA cluster in DLK1-DIO3 genomic region, which plays a role in several pathologic processes of various cancers, especially bladder cancer. Li et al. showed that the methylation of DLK1-MEG3 intergenic DMR contributed to the decrease in levels of serum miR-323a-3p in bladder cancer. The progression of EMT was suppressed through miR-323a-3p/MET/SMAD3/SNAIL circuit (Li et al., 2017). A study elucidated the existence of mutual regulation of BCa progression involving miR-323a-3p/miR-433/miR-409 and MET. The study investigated the role of miR-433 and miR-409 in the development and progression of BCa. The results showed that miR-409-3p could serve as metastasis-suppressor gene by directly targeting c-Met and indirectly regulating MMP2 and MMP9 (Xu et al., 2013). CAMP response element-binding protein1 (CREB1) and c-Met could take part in miR-433-mediated inhibition of the EMT by regulating Akt/GSK-3β/Snail signaling. Also, a reciprocal regulation network between miR-433/miR-409-3p and c-Met was revealed (Xu et al., 2016). These findings might be useful in the search for effective and promising therapies against BCa.

### Colorectal Cancer (CRC)

CRC is a public health concern and accounts for over 10.2% of all cancer cases, representing 1.8 million new cases per year (Bray et al., 2018). Despite widespread awareness in the general population regarding CRC, the condition remains a significant cause of mortality and morbidity on account of recurrence and metastasis.

Studies have confirmed that the expression of c-Met in CRC correlates with the presence of local and distal metastasis and poor prognosis. Congruently, Migliore et al. showed that miR-1 is downexpressed in colon cancer cells, and is significantly associated with MET overexpression, especially in metastatic tumors. Further analysis revealed that concomitant MACC1 (MET transcriptional activator) upregulation and miR-1 downregulation are required to elicit the highest level of MET expression. Induced miR-1 expression reduces MET levels and impairs MET-induced proliferation, migration and invasion processes. Notably, a feedback loop between miR-1 and MET has also been revealed. Most likely, MET targeting could result in both abrogations of MET-dependent signaling and some recovery of miR-1 level (Migliore et al., 2012). Siemens verified that the generation of distant metastases is correlated with epigenetic silencing of miR-34a in primary tumors. In their study, they reported that miR-34a expression was inversely correlated with CpG methylation and its targets, that is, c-Met, Snail, and β-catenin, and were associated with distant metastases. In the same study, a multivariate regression model containing miR-34a methylation, high c-Met, and β-catenin levels indicated high prognostic value of CRC cells' metastasis to the liver (Siemens et al., 2013). In 2018, Bleau et al. identified c-Met as a prometastatic gene and miR-146a as a negative regulator of c-Met in highly metastatic variants derived from MC38 cells. Further, they firstly confirmed that miR-146a has a crucial inhibitory role in liver metastasis and this suppressive effect is probably because of targeting several protumorigenic genes, including c-Met (Bleau et al., 2018).

Further, the ceRNA network has also been reported to play a crucial role in CRC progression. The study results showed that lncRNA GAPLINC was upregulated in CRC tissues and thus stimulated CRC cell migration and invasion by regulating miR-34a/c-MET signal pathway (Luo et al., 2018). Analogously, lncRNA 01510 was reported that its overexpression was associated with advanced clinicopathological features. Meanwhile, Cen et al. also found that silencing LINC01510 could restrain cell proliferation and induce G0/G1-phase arrest via a MET-dependent manner (Cen et al., 2018). In 2019, Zhong et al. constructed the lncRNA/pseudogene–miRNA–mRNA ceRNA network using The Cancer Genome Atlas database and verified that lncRNA H19, which was upregulated in CRC tissue and correlated with poor prognosis, was positive related to MET expression. However, the specific molecular mechanism and function were not explored (Zhong et al., 2019). These findings provided substantial evidence that c-Met has excellent potential as an effective agent for both the prevention and treatment of colorectal cancer.

### Brain Cancer

Glioma is the most common malignancy of the central nervous system and is associated with a poor prognosis. Several studies suggest that the condition could occur as a result of complicated gene interaction and molecular modulation network (Sturm et al., 2017). *In vitro* and xenograft model experiments have verified that miR-410 repression attenuates tumor growth and invasion by inhibiting MET and AKT signaling. Also, MET overexpression could significantly abrogate miR-410 dependent effects on glioma proliferation and invasion (Chen L. et al., 2012). With regard to lncRNA, Li et al. found that lncRNA NEAT1was overexpressed in glioma tissue and cell lines and served as a ceRNA to promote glioma pathogenesis. Moreover, lncRNA NEAT1's oncogenic activity was enhanced through inverse regulation of miR-449b-5p and c-Met modulation in glioma cells (Zhen et al., 2016). This was the first report on the role and function of lncRNA NEAT1 in glioma.

Concerning glioblastoma (GBM), it has been reported that transient transfection of miR-34a mimics into glioma and medulloblastoma cell lines remarkably inhibits cell proliferation, invasion, survival, and cell cycle progression by targeting c-Met, Notch-1, and Notch-2 (Li Y. et al., 2009). MiR-144-3p was downregulated in GBM tissue, and this decrease was significantly correlated with ascending grades and poorer overall survival. Exogenetic miR-144-3p expression compromised the malignant biological properties of GBM cells independent of PTEN and resulted in enhancement of radiation and temozolomide (TMZ) sensitivity by binding to c-Met and thus impairing the activity of downstream signaling (Lan et al., 2015). As for lncRNAs, Wu et al. firstly identified that lncRNA TALC, located on the AL358975 locus and consisted of two exons with a full length of 418 nt, had elevated expression in temozolomide-resistant GBM cells. Further mechanism exploration revealed that lncRNA TALC induced TMZ resistance in GBM through trapping miR-20b-3p and activating c-Met. Meanwhile, lncRNA TALC also upregulated MGMT expression by refitting the acetylation of H3K9, H3K27, and H3K36 in MGMT promoter regions via c-Met/Stat3/p300 axis (Wu et al., 2019). Despite the identification of many new biomarkers for brain cancer, improvement in treatment options is yet to be achieved.

### Female Reproductive Cancers

One of the most prevalent cancers of the female reproductive system is ovarian cancer (Eisenhauer, 2017). Substantial evidence shows that some miRNAs (such as miR-1, miR-206, and miR-148a-3p) can suppress the proliferation, survival, invasion, and metastasis of ovarian cancer cells by directly targeting c-Met, and subsequently regulating downstream signaling pathway to serve as tumor suppressors such as PI3K/Akt/mTOR and ERK1/2 pathways (Qu et al., 2017; Dai et al., 2018; Wang W. et al., 2018). As for lncRNAs, Qiu et al. highlighted the role of lncRNA antisense non-coding RNA in the INK4 locus (ANRIL) in serous ovarian cancer (SOC). They demonstrated its elevated level in SOC tissue and cell lines, particularly in high metastatic cell lines. Meanwhile, there was significant association between lncRNA ANRIL expression and advanced FIGO stage, high histological grade, lymph node metastasis, and poor prognosis. Moreover, MET and MMP3 were verified as key downstream genes of ANRIL involved in SOC cell migration/invasion by microarray analysis and Western Blotting (Qiu et al., 2015). Therefore, a breakthrough in ovarian cancer treatment could be achieved by a combination of therapy involving both ncRNAs and c-Met inhibitors.

Cervical cancer is a frequent aggressive malignancy and the fourth leading cause of cancer-related deaths among females worldwide, particularly in China. MiR-454-3p mimics treatment has been shown to suppress the proliferation, migration, and invasion ability of cervical tumor cells *in vitro* by targeting c-Met (Guo et al., 2018). The downregulation of miR-1 in cervical cancer tissues is correlated with high c-Met expression. Also, c-Met upregulation inhibits E-cadherin expression, which triggers the proliferation, migration, and infiltration of cancer cells, thus lowering the survival rates of the patient (Cheng Y. et al., 2019). In addition, ceRNA also has been reported in cervical cancer. LncRNA SNHG4 was overexpressed in cervical cancer tissue and cells. And this upregulation could facilitate proliferation and reduce apoptosis by binding to miR-148a-3pand ultimately activating c-Met (Li et al., 2019). These findings indicate that c-Met isan attractive therapeutic target for cervical cancer.

Endometrial cancer (EC) is the most epidemic neoplasm of the female genital tract worldwide. Despite the great development in early identification and treatment, an abundant number of cases of advanced ECs are still diagnosed (Herrero et al., 2019). LncRNA SNHG8, which are located on Chr4, has been verified to participate in the progression and drug resistance of multiple malignancies, such as esophageal squamous cell carcinoma (Song et al., 2019), gastric cancer (Zhang P. et al., 2019), pancreatic adenocarcinoma (Song et al., 2018), and hepatocellular carcinoma (Dong et al., 2018). In 2018, Yang et al. firstly verified the positive association between lncRNA SNHG8 and c-Met in EC. They found silencing lncRNA SNHG8 resulted in weaker c-MET expression and less proliferation. Meanwhile, this reduction could be reversed by addition of miR-152 inhibitor (Yang C. H. et al., 2018). This study connected ncRNA with HGF/c-Met axis together in EC and demonstrated the significant importance of HGF/c-Met axis in EC.

### Pancreatic Cancer

Pancreatic cancer is one of the most aggressive malignancies, and is the seventh cause of cancer-related mortality worldwide, with a 5-year survival rate of about 5% (Bray et al., 2018). Tomihara et al. were the first to confirm the association between preoperative chemoradiation therapy and c-Met expression in pancreatic ductal adenocarcinoma (PDAC). They successfully confirmed the association between irradiation-induced c-Met expression and activated c-Met pathways through miR-181b-5p/ETS1/c-Met axis in PDAC cells (Tomihara et al., 2017). Recently, circRNA and exosomes were shown to play essential roles in various tumors, particularly PDAC. Circ-PDE8A, a highly contained and stable circular RNA screened out from tumor exosomes, is overexpressed in PDAC tissues and is an independent risk factor for PDAC survival. Its upregulation could stimulate tumor migration and growth through miR-338/MACC1/MET/AKT or ERK pathway by sponging miR-338. Further, they found that circ-PDE8A excreted by tumor could be released into blood circulation through exosome transportation. Also, plasma exosomal circ-PDE8A was correlated with PDAC invasion and prognosis (Li Z. et al., 2018). Modali et al. demonstrated the importance of HGF/c-Met axis in development of pancreatic neuroendocrine tumors. They revealed that menin could activate lncRNA Meg3 through H3K4me3 and CpG hypomethylation. And lncRNA Meg3 was shown to have tumor suppressor activity because of its role in suppression of cell growth and cycle progression by negatively targeting c-Met (Modali et al., 2015). All these findings provided a strong basis for considering c-Met suppression as novel therapeutic approach for the treatment of pancreatic cancer through prevention of metastasis and irradiation resistance.

### Prostate Cancer

Prostate cancer accounts for 3.8% of cancer-related deaths annually, and this has economic implications on the public health sector worldwide (Bray et al., 2018). Treatment of the condition using exogenous miR-34c and miR-1 has been shown to suppress MET expression by preventing its binding to 3′-UTR and thereby inhibiting prostate cancer's aggressive phenotype (Hagman et al., 2013; Gao et al., 2019). Further studies have confirmed that miR-1 can suppress c-Met/PI3K/Akt/mTOR signaling and impair tumor progression (Gao et al., 2019). However, the specific mechanism of miR-34c is still unclear.

### Other Cancers

Oral squamous cell carcinoma (OSCC) represents a unique and major health concern all over the world. A study by Xu et al. indicated that miR-143 inhibited OSCC cell mobility by suppressing phospho-c-Met expression through targeting CD44 v3 (Xu et al., 2015). The miR-23b/27b cluster was also shown to inversely regulate MET in OSCC and bladder cancers (Fukumoto et al., 2016). Analogously, miR-152 served as a tumor suppressor by directly targeting c-Met (Li M. et al., 2018). Moreover, Huang et al. unveiled a novel c-Met regulating mechanism that might be applied as a modality for OSCC therapy. They demonstrated that miR-365-3p/ETS homologous factor (EHF, a KRT16transcription factor) axis could reduce OSCC cell migration, invasion, metastasis, and chemoresistance to 5-fluorouracil through the suppression of KRT16. In the same study, depletion of KRT16 resulted in ample protein degradation of β5-integrin and c-Met via a lysosomal pathway, subsequently led to the repression of downstream Src/STAT3/FAK/ERK signaling in OSCC cells (Huang et al., 2019). Generally, c-Met plays a crucial role in OSCC progression and could be a potential target in OSCC therapy.

In renal cell carcinoma (RCC), miR-199a-3p is known as a tumor attenuator. Huang et al. revealed that miR-199a-3p was downregulated in RCC primary tumor and cell lines, and this loss was associated with advanced tumor-lymph node-metastasis (TNM) stage and Fuhrman grade. Reintroducing miR-199a-3p in RCC cell lines inhibited proliferation and led to the arrest ofG1 phase by negatively regulating c-Met, thus suppressing HGF/c-Met axis and it's downstream signaling such as Akt, ERK1/2 and mTOR (Huang et al., 2014). These provided a potential target for RCC. Wang et al. reported that miR-32-5p functions by altering the TR4/HGF/Met/MMP2-MMP9 signaling to suppress the clear cell renal cell carcinoma (ccRCC) metastasis *in vitro* and *in vivo*. Also, they demonstrated that targeting miR-32-5p/TR4/HGF/Met signaling could enhance the efficacy of current sunitinib-chemotherapy for ccRCC suppression (Wang M. et al., 2018). For lncRNA, Qu et al. first identified a novel lncRNA, called lncARSR (lncRNA Activated in RCC with Sunitinib Resistance), which could promote sunitinib resistance and predict poor response of RCC patients. Moreover, they found that intercellular transfer of lncARSR by exosomes disseminated sunitinib resistance. Mechanically, lncARSR functioned as a ceRNA for miR-34 and miR-449 to promote AXL and c-MET expression (Qu et al., 2016). In addition, overexpression of lncRNA NEAT1 was also observed in RCC cells, and its elevated levels were associated with poor prognosis. The silencing of lncRNA NEAT1 has been shown to inhibit RCC cell proliferation, migration, and invasion by inhibiting cell cycle progression and EMT phenotype. Down-regulation of lncRNA NEAT1 enhanced the sensitivity of RCC cells to sorafenib *in vitro*. Mechanistic analysis revealed that lncRNA NEAT1 acts as a competitive sponge for miR-34a, therefore preventing inhibition of c-Met (Liu F. et al., 2017). Therefore, ncRNAs and c-Met might serve as a predictor and a potential therapeutic target for RCC.

In thyroid cancer (TC), the crosstalk between ncRNAs and HGF/c-Met axis has a significant effect on tumor growth and progression. Several reports have shown that some miRNAs (such as miR-449b and miRNA-3666) act as tumor suppressors that inhibit TC growth and reduce apoptosis by decreasing the levels of MET protein rather than its transcription (Chen L. et al., 2015; Wang et al., 2016). Moreover, ceRNA plays a role in thyroid cancer progression. Liu et al. also observed a significant increase in lncRNA XIST expression in thyroid cancer tissues and cell lines. Currently, lncRNA XIST is used as an important prognostic predictor for TC patients. Further investigation involving *in vitro* and *in vivo* experiments, online datasets, and online predicting tools has revealed that lncRNA XIST serves as a ceRNA for miR-34a by sponging miR-34a, and competing with MET for miR-34a binding, hence modulated thyroid cancer cell proliferation and tumor growth (Liu et al., 2018). These findings provided a foundation for TC therapy improvement based on lncRNA-miRNA-mRNA interaction.

Esophageal squamous cell carcinoma (ESCC) is one of the major histological subtypes of esophageal cancer in China. Currently, there are limited therapeutic options for the disease because of limited understanding of its molecular mechanism. MiR-1 functions as a crucial tumor inhibitor in ESCC (same as its role in HCC, GC, and ovarian cancer). Its overexpression is associated with a decrease in acquisition of MET, cyclin D1, and CDK4 (oncogene and cell cycle-related proteins), which eventually lead to are duction in cell growth and enhanced cell apoptosis *in vitro* and *in vivo* (Jiang et al., 2016). The downregulation of miR-206 is associated with ESCC of lymph node metastasis, advanced TNM stage, and overall survival. Furthermore, luciferase reporter gene assay revealed that c-Met is a direct target of miR-206, and ectopic miR-206 expression inhibits ESCC cell proliferation and induces apoptosis by inhibiting c-Met/AKT/mTOR pathway (Zhang J. et al., 2019). Collectively, these findings confirm the role of c-Met in ESCC progression.

Multiple myeloma (MM) is the second most general hematological cancer in globe with poor survival on account of high recurrence (Chen et al., 2019). In 2019, Yang and his team demonstrated that HGF/c-Met axis played an essential role in MM development. They first verified the overexpression of lncRNA UCA1 in MM and clarified its tumor-promoting effect (mainly facilitating proliferation and reducing apoptosis) could be regulated by miR-1271-5p/HGF axis (Yang and Chen, 2019). These indicated that RNA regulation might be a new target for the cure of MM.

Hepatoblastoma (HB) is a highly invasive neoplasm in childhood. In 2019, Cui et al. revealed a vital role of crosstalks between ncRNAs and HGF/c-Met axis in HB. LncRNA ZFAS1 is a snoRNA host gene locating in chromosome 20q13.13. They found that lncRNA ZFAS1 was overexpression in HB tissue and correlated with malignant pathological features and poor prognosis through tissue microarray analysis. Furthermore, they also showed that lncRNA ZFAS1 served as a ceRNA to regulate RALY by sponging miR-193a-3p and played an oncogenic role during HB progression via HGF/c-Met pathway during HB development (Cui et al., 2019). This study expounded a specific molecular mechanism of HB development involving ZFAS1/miR-193a-3p/RALY axis, which could be functioned as a potential biomarker and therapeutic target for HB.

As for retinoblastoma, Yang and his team found that there was a negative correlation between the expression of lncRNA HOTAIR and miR-613 in retinoblastoma tissue compared with normal tissue. And lncRNA HOTAIR could stimulate progression and aggravation of retinoblastoma by miR-613/c-Met axis (Yang G. et al., 2018). Although this study provided great evidences for identifying potential diagnostic and therapeutic target in retinoblastoma, further exploration was in demand.

Diffuse large B-cell lymphoma (DLBCL) is the most frequent lymphoid malignancy all over the world. MET was reported to be upregulated in DLBCL and predicted poor outcomes. Cheng et al. investigated that elevated lncRNA TUG1 expression could facilitate cell proliferation and decrease apoptosis *in vitro* and *in vivo* by increasing MET expression through inhibiting its ubiquitination and degradation (Cheng H. et al., 2019). In summary, this study provided a novel insight for DLBCL treatment.

## Conclusions and Future Perspectives

In this review, we have summarized the crosstalk between HGF/c-Met axis and ncRNAs in common multiple human cancers, which has improved our understanding on the pathogenetic mechanism of tumor occurrence and development. However, further studies are required to fully explore the interaction between ncRNAs and HGF/c-Met axis in cancer. Despite the evidence presented here, the precise interactions between ncRNAs and HGF/c-Met axis, and how these interactions affect tumorigenesis and progression of cancers are still largely unknown. This implies that additional controlled and large-scale clinical studies are required before cancer-specific ncRNAs and HGF/c-Met inhibitors can be recommended for clinical diagnosis and treatment.

Taken together, deeper understanding of HGF/c-Met axis will reveal beneficial avenues and generate new hypotheses regarding cancer pathogenesis and treatment.

## Author Contributions

ZY, ZR, and RS proposed the study. XL, JC, LL, XC, SS, and GC performed the research and wrote the first draft. ZR and ZY are the guarantors. All authors contributed to interpretation of the study and to further drafts.

### Conflict of Interest

The authors declare that the research was conducted in the absence of any commercial or financial relationships that could be construed as a potential conflict of interest.

## Abbreviations

HGF: hepatocyte growth factor
c-Met: c-mesenchymal-epithelial transition factor
EMT: epithelial-mesenchymal transition
ncRNAs: non-coding RNAs
NSCLC: non-small cell lung cancer
HCC: hepatocellular carcinoma
GC: gastric cancer
DDP: cisplatin
ceRNAs: competing endogenous RNAs
OS: osteosarcoma
BCa: bladder cancer
CRC: colorectal cancer
GBM: glioblastoma
TMZ: temozolomide
SOC: serous ovarian cancer
EC: endometrial cancer
OSCC: oral squamous cell carcinoma
RCC: renal cell carcinoma
ccRCC: clear cell renal cell carcinoma
PDAC: pancreatic ductal adenocarcinoma
PNET: pancreatic neuroendocrine tumors
TC: thyroid cancer
ESCC: esophageal squamous cell carcinoma
MM: multiple myeloma
HB: hepatoblastoma
DLBCL: diffuse large B-cell lymphoma.
